# CSFV induced mitochondrial fission and mitophagy to inhibit apoptosis

**DOI:** 10.18632/oncotarget.17030

**Published:** 2017-04-11

**Authors:** Hongchao Gou, Mingqiu Zhao, Hailuan Xu, Jin Yuan, Wencheng He, Mengjiao Zhu, Hongxing Ding, Lin Yi, Jinding Chen

**Affiliations:** ^1^ College of Veterinary Medicine, South China Agricultural University, Guangzhou, People's Republic of China

**Keywords:** classical swine fever virus (CSFV), mitochondrial fission, mitophagy, apoptosis

## Abstract

Classical swine fever virus (CSFV), which causes typical clinical characteristics in piglets, including hemorrhagic syndrome and immunosuppression, is linked to hepatitis C and dengue virus. Oxidative stress and a reduced mitochondrial transmembrane potential are disturbed in CSFV-infected cells. The balance of mitochondrial dynamics is essential for cellular homeostasis. In this study, we offer the first evidence that CSFV induces mitochondrial fission and mitophagy to inhibit host cell apoptosis for persistent infection. The formation of mitophagosomes and decline in mitochondrial mass relevant to mitophagy were detected in CSFV-infected cells. CSFV infection increased the expression and mitochondrial translocation of Pink and Parkin. Upon activation of the PINK1 and Parkin pathways, Mitofusin 2 (MFN2), a mitochondrial fusion mediator, was ubiquitinated and degraded in CSFV-infected cells. Mitophagosomes and mitophagolysosomes induced by CSFV were, respectively, observed by the colocalization of LC3-associated mitochondria with Parkin or lysosomes. In addition, a sensitive dual fluorescence reporter (mito-mRFP-EGFP) was utilized to analyze the delivery of mitophagosomes to lysosomes. Mitochondrial fission caused by CSFV infection was further determined by mitochondrial fragmentation and Drp1 translocation into mitochondria using a confocal microscope. The preservation of mitochondrial proteins, upregulated apoptotic signals and decline of viral replication resulting from the silencing of Drp1 and Parkin in CSFV-infected cells suggested that CSFV induced mitochondrial fission and mitophagy to enhance cell survival and viral persistence. Our data for mitochondrial fission and selective mitophagy in CSFV-infected cells reveal a unique view of the pathogenesis of CSFV infection and provide new avenues for the development of antiviral strategies.

## INTRODUCTION

Classical swine fever virus (CSFV), which is associated with hepatitis C and dengue virus, is an enveloped RNA virus that belongs to the *Pestivirus* within the family *Flaviviridae* [1, 2]. The single positive-stranded genome of CSFV contains a unique large open reading frame encoding a polyprotein that is subsequently processed into 12 known proteins by cellular and viral proteases: N^pro^, C, E^rns^, E1, E2, p7, NS2, NS3, NS4A, NS4B, NS5A and NS5B [3–5]. Different pathological changes are observed in pigs infected with strains of varied virulence. Highly virulent strains, such as the shimen strain, induce acute progression with high mortality rates and typical clinical characteristics including hemorrhagic syndrome and immunosuppression, while strains of low-to-moderate virulence can persist *in vivo* with no obvious appearance [3, 6–10]. The complex interplay between CSFV and the host makes it difficult to eliminate [11]. Thus, classical swine fever (CSF), the economically important animal disease worldwide, has been listed as A by the OIE (World Organisation for Animal Health) [12]. Interestingly, no cytopathic effect is apparent when CSFV reproduces in host cells *in vitro* [13, 14]. Although many studies related to the mechanism of CSFV replication have been performed, the pathogenesis of this virus is still poorly understood [15–17].

Mitochondria, which are organelles with outer (OMM) and inner membrane bilayers, participate in a wide variety of crucial cellular processes such as ATP production, apoptosis, calcium homoeostasis, cellular proliferation, and the synthesis of amino acids, nucleotides, and lipids [18, 19]. Under extrinsic and intrinsic stimuli, mitochondrial quality control, including fission, fusion, and selective autophagic degradation of mitochondria (mitophagy), are necessary for cell viability and bioenergetics [20]. A number of viral proteins target to mitochondria and interact with mitochondrial proteins, resulting in ROS accumulation, mitochondrial Ca^2+^ overload, the collapse of mitochondrial transmembrane potential, and subsequent mitochondrial dysfunction [21–25]. Notably, several viruses such as hepatitis C virus, hepatitis B virus and influenza A virus can trigger virus-specific mitophagy to balance aberrant mitochondrial dynamics [26–31].

Mitophagy is a well-studied type of mitochondrial degradation process. Unlike non-selective autophagy, mitophagy occurs independently after selective recognition of damaged or excessive mitochondria by some special receptors [32]. Recent work has linked defects in Pink1-Parkin signaling pathway-mediated mitophagy priming to Parkinson's disease [33–35]. Parkin is an E3 ubiquitin ligase with a widespread physiological role [36]. Once mitochondrial stress is induced, it rapidly translocates from the cytosol to depolarized mitochondria [37–39]. PINK1, an OMM Ser/Thr kinase, can regulate and facilitate Parkin targeting of the damaged mitochondria [40–42]. Although the role of mitophagy in viral infections is now becoming clarified, the function of Parkin in virus-induced mitophagy is still fraught with controversy [27, 30, 43].

CSFV has been shown to induce oxidative stress in porcine umbilical vein endothelial, macrophage and kidney cell lines [44–46]. *In vivo*, a reduced mitochondrial transmembrane potential and mitochondrial dysfunction in apoptotic lymphocytes has been detected [10]. Our previous work has confirmed that CSFV coordinates the autophagy pathway (macro-autophagy) to enhance viral replication and release in host cells [47]. In this study, we investigated Parkin-mediated mitophagy in replicating CSFV cells. Abnormal mitochondria were observed in CSFV-infected cells using electron microscopy. The decline in mitochondrial mass was linked to CSFV infection and prevented by using autophagy inhibitors. We found that the expression and mitochondrial translocation of PINK1 and Parkin were increased by CSFV infection, and the degradation of Mitofusin2 (MFN2) via the ubiquitin pathway was upregulated in CSFV-infected cells. Meanwhile, formation of the mitophagosome and mitophagolysome, respectively, induced by CSFV infection were manifested by confocal analysis of GFP-LC3-targeting mitochondria merged with Parkin and lysosomes. In addition, we employed a dual fluorescence reporter, Mito-mRFP-EGFP, to demonstrate the delivery of mitochondria to lysosomes. Furthermore, mitochondrial fission in CSFV-infected cells was explored by observing the translocation of Drp1 to mitochondria by confocal microscopy. Finally, silencing of Drp1 or Parkin in CSFV-infected cells revealed an effect of mitochondrial fission or mitophagy on CSFV replication and apoptosis. In summary, our results suggested that CSFV triggered mitochondrial fission and Parkin-mediated mitophagy to maintain the cell viability of host cells and promote persistent viral infection.

## RESULTS

### The decline in mitochondrial mass in CSFV-infected cells was related to mitophagy

**Figure 1:**
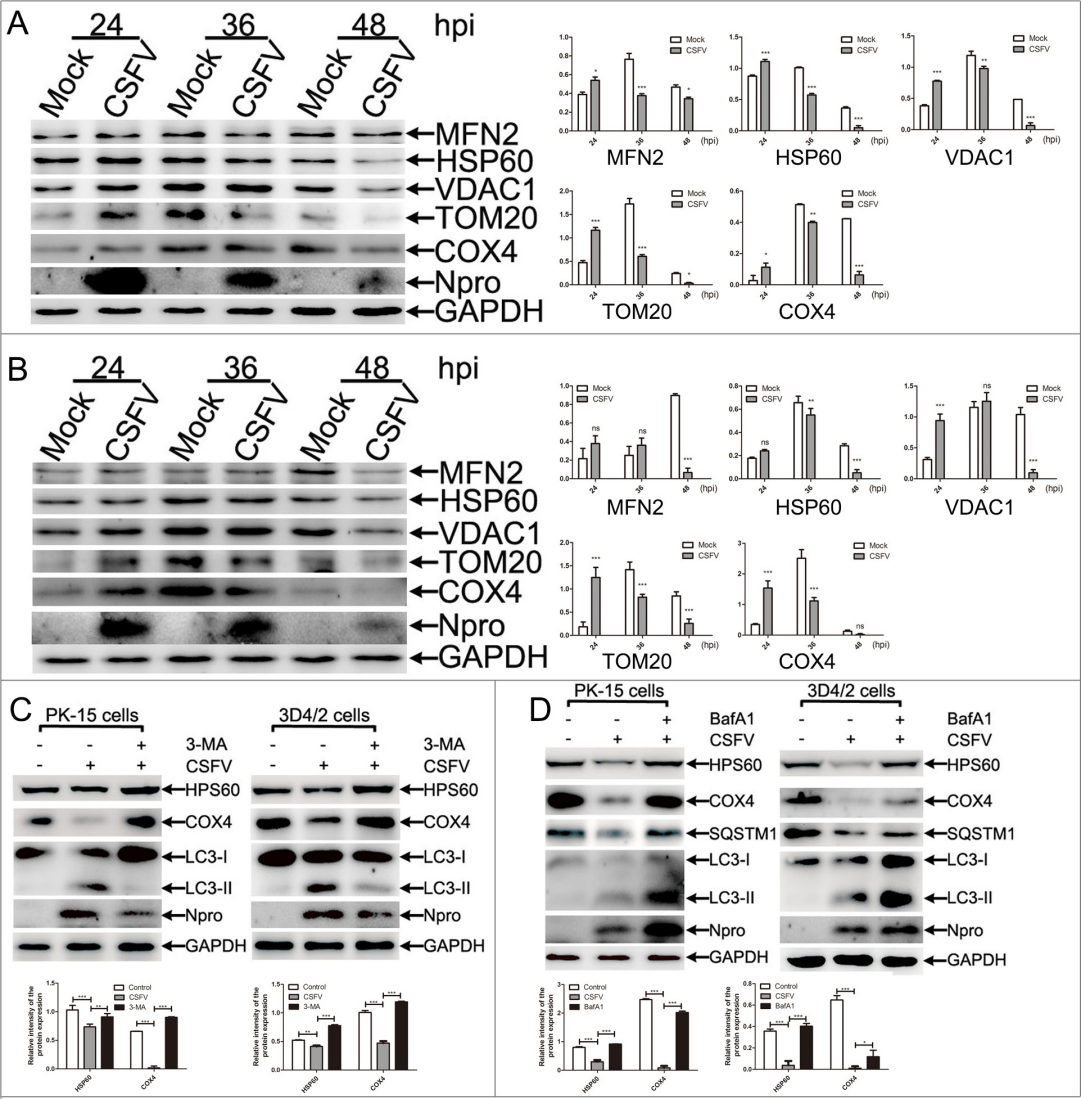
The decline in number of mitochondria in CSFV-infected cells was related to mitophagy **(A)** Changes of mitochondrial proteins in CSFV-infected PK-15 cells. PK-15 cells were mock-infected or infected with CSFV (MOI = 1), and whole cell lysates (WCL) were prepared at 24, 36 and 48 hpi. The expression of mitochondrial proteins, including MFN2, HSP60, VDAC1, TOM20 and COX4, were analyzed by Western blotting. CSFV infection was verified by immunoblotting with anti-CSFV Npro antibody. GAPDH was used as an internal loading control. The histograms on the right showed the statistical analysis of the protein band intensity (mean ± SD; n = 3; *P < 0.05; ^*^P< 0.01; ^**^*P < 0.001). P values were calculated using two-way ANOVA. **(B)** Changes of mitochondrial proteins in CSFV-infected 3D4/2 cells were analyzed as in **(A)**. **(C)** Changes of mitochondrial proteins in CSFV-infected PK-15 and 3D4/2 cells treated with 3-MA. PK-15 and 3D4/2 cells infected with CSFV (MOI = 1) in the presence or absence of 3-MA (5 mM) at 48 hpi. Expression of mitochondrial matrix proteins including HSP60 and COX4 were by Western blotting. Inhibition of autophagy determined by the detection of LC3-II expression. CSFV infection was verified by immunoblotting with anti-CSFV Npro antibody. GAPDH was used as an internal loading control. The lower histograms showed the statistical analysis of the intensity of mitochondrial protein bands (mean ± SD; n = 3; ^*^P< 0.01; ^**^*P < 0.001). P values were calculated by two-way ANOVA. **(D)** Changes of mitochondrial proteins in CSFV-infected PK-15 and 3D4/2 cells treated with BafA1. PK-15 and 3D4/2 cells were infected with CSFV (MOI = 1) in the presence or absence of BafA1 (5 nM) at 48 hpi. HSP60 and COX4 were analyzed as in **(C)**. Inhibition of the autophagy flux was evaluated by detecting LC3-II and SQSTM1. The lower histograms showed the statistical analysis of the intensity of mitochondrial protein bands (mean ± SD; n = 3; *P< 0.5; ^**^*P < 0.001). P values were calculated by two-way ANOVA.

Mitophagy is usually related to a decline in mitochondria mass, and the expression of mitochondrial proteins analyzed by Western blotting is a common way to reflect the mitochondrial mass. An analysis of mitochondrial matrix proteins would be relatively reasonable for evaluating mitophagy because of the possibility that outer mitochondrial membrane proteins such as TOM20, VDAC, and MFN1/2 might be degraded by the proteasome [32]. To assess CSFV-induced mitophagy, we first evaluated changes in mitochondrial proteins in PK-15 and 3D4/2 cells-infected by CSFV at different hours post-infection (hpi). As shown in Figure 1A and 1B, both the expression of outer mitochondrial membrane proteins (TOM20, VDAC1 and MFN2) and mitochondrial matrix proteins (HSP60 and COX4) showed a decreased tendency after 36 hpi, indicating that the mitochondrial mass was downregulated by CSFV infection. Interestingly, we found that Npro protein displayed reversed levels compared with increased viral replication from 24 to 48 hpi (Figure 1A, 1B and Supplementary Figure 1). This phenomenon may be related to rapid proteasomal degradation of Npro protein in cell culture [48]. To validate that the decline in mitochondrial mass was related to mitophagy, we treated PK-15 and 3D4/2 cells with autophagy or lysosome inhibitors before and during CSFV infection. Both 3-methlyadenine (3-MA) inhibition of autophagic phagophore formation and Bafilomycin A1 (BafA1) inhibition of the activity of vacuolar-type H^+^-ATPase could invert the degradation of mitochondrial matrix proteins induced by CSFV infection. Moreover, LC3-II and SQSTM1 monitoring of the effect of inhibitors displayed the corresponding changes (Figure 1C and 1D).

### The ultrastructure of mitochondria in CSFV-infected cells was altered

**Figure 2:**
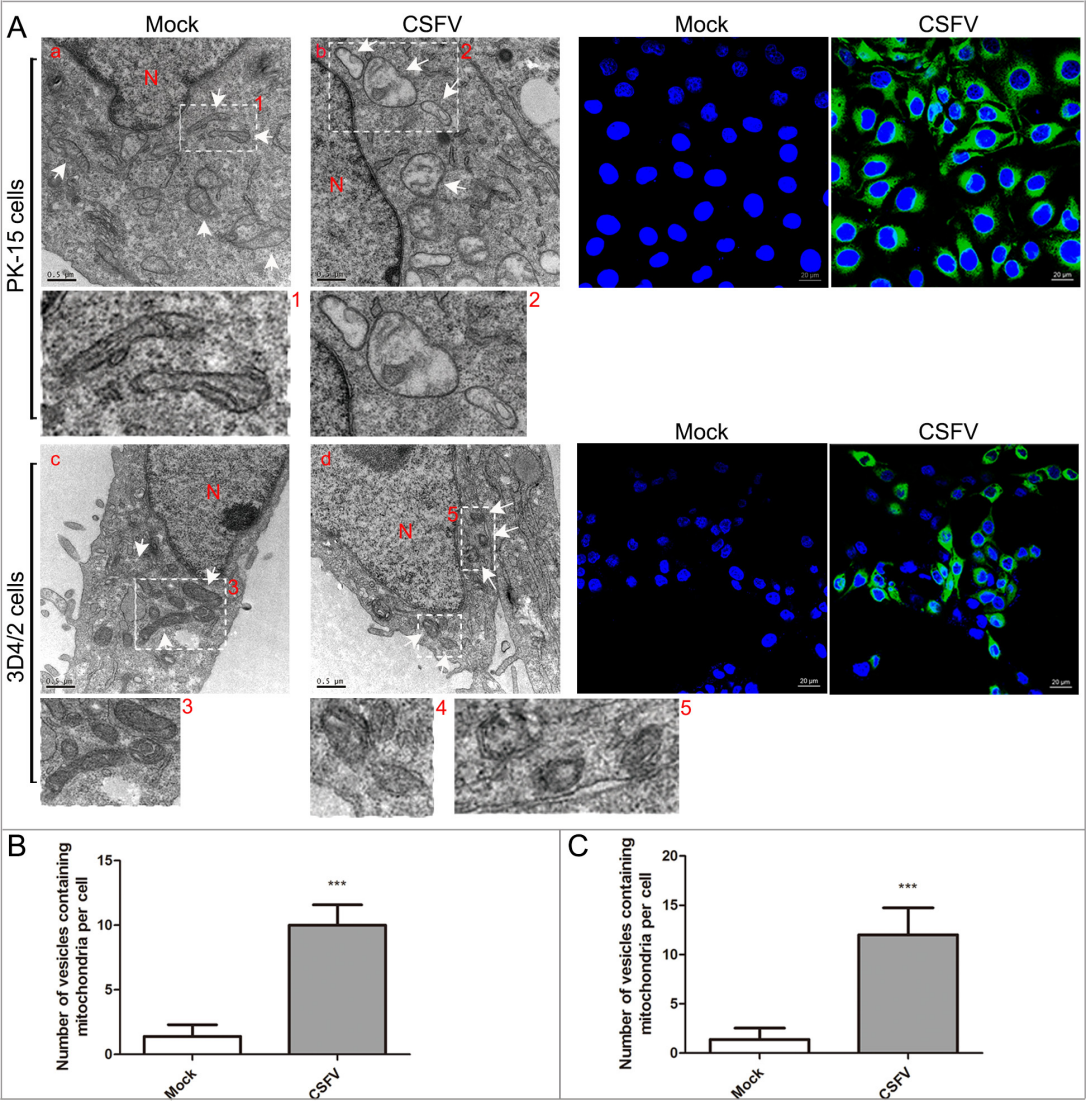
CSFV infection induced abnormal mitochondria and the formation of mitophagosomes in PK-15 and 3D4/2 cells **(A)** Electron microscopy images revealed the mitochondrial ultrastructure in CSFV-infected cells. PK-15 **(a** and **b)** and 3D4/2 **(c** and **d)** cells were mock-infected or CSFV-infected at an MOI of 1 for 48 h and analyzed by electron microscopy. In the zoomed images, typical elongated tubular mitochondria in uninfected cells and fragmented elliptic mitochondria engulfed with membrane-like vesicles in CSFV-infected cells were shown. Organelle mark: N, nucleus; White arrow, normal mitochondria or mitophagosomes. Scale bar = 0.5 mM. Confocal images indicate the ratio of cells infected by CSFV. Cells were immunostained with anti-CSFV E2 antibody (green). Nuclei were stained with DAPI (blue). **(B** and **C)** Quantification of the mitophagosome-like vesicles per PK-15 **(B)** and 3D4/2 **(C)** cell image (mean ± SD; n ≥ 5 cells; ***P < 0.001). P values were calculated using an unpaired Student's t-test.

In addition to autophagy, transmission electron microscopy (TEM) is still considered one of the gold standards providing important evidence of mitophagy [32]. To examine whether CSFV can induce mitophagy, we observed the ultrastructure of mitochondria in PK-15 and 3D4/2 cells infected with CSFV at 48 hpi. Confocal images showed that most cells in the CSFV-infected group were infected (Figure 2A). TEM images showed elliptic mitochondria with a dramatic loss of mitochondrial cristae in CSFV-infected cells (Figure 2A, 2A-b and 2A-d). Notably, CSFV infection resulted in an increased number of mitochondria that were trapped by double or single membrane vesicles in CSFV-infected cells (Figure 2A-2, 2A-4 and 2A-5). Quantitative analysis also showed a significant increase in the quantity of mitophagosome-like structures in CSFV-infected cells (Figure 2B and 2C). In contrast, the tubular mitochondrial morphology with typical cristae was inspected in cells of the mock group (Figure 2A, 2A-a and 2A-c), and mitochondria engulfed by membrane vesicles were rarely found. Changes in mitochondrial ultrastructure in CSFV-infected cells underscored the CSFV-disrupted dynamics of cellular mitochondria.

### CSFV infection enhanced mitochondrial recruitment of Parkin and ubiquitination of its mitochondrial substrate MFN2

**Figure 3:**
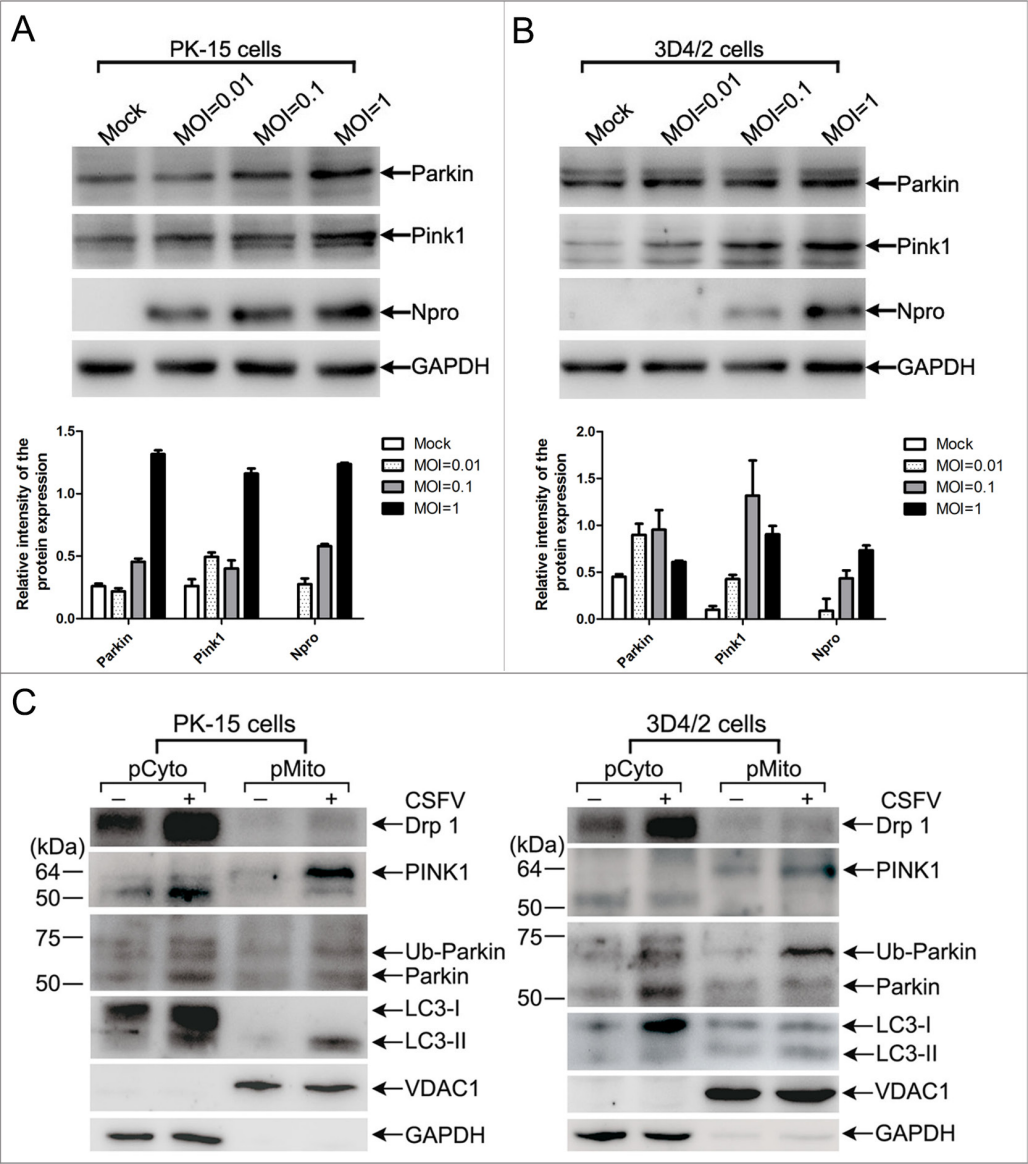
PINK1-Parkin pathway was activated by CSFV infection **(A** and **B)** Expression of PINK1 and Parkin in CSFV-infected cells. PK-15 **(A)** and 3D4/2 **(B)** cells were mock-infected or infected with CSFV (MOI = 0.01, 0.1 and 1), respectively. PINK1 and Parkin expression were analyzed by Western blotting at 48 hpi. CSFV infection was verified by immunoblotting with anti-CSFV Npro antibody. GAPDH was used as an internal loading control. (Lower histograms, the relative intensity of Parkin, PINK1 and Npro expression normalized to GAPDH was analyzed by Image-pro plus 6.0, mean ± SD; n = 3); **(C)** The translocation of mitophagy-related proteins to mitochondria in CSFV-infected cells. PK-15 and 3D4/2 cells were mock-infected or infected with CSFV (MOI = 1). At 48 hpi, pure cytoplasm and mitochondrial fractions were isolated as described in Materials and Methods. The indicated proteins in fractions were analyzed by immunoblotting using specific antibodies. Fractions: purified cytoplasm, pCyto; purified mitochondria, pMito. organelle markers: VDAC1, mitochondria; GAPDH, cytoplasm.

The molecular mechanisms of mitophagy only begin to be characterized in mammalian cells, and the PINK1-Parkin signaling pathway is well-studied among multiple molecular regulators. The E3 ubiquitin ligase Parkin can selectively translocate to depolarized mitochondria, resulting in mitophagy after activation by the kinase PINK1 [38, 40]. Therefore, we set out to address whether CSFV might have an influence on the regulation of PINK1 and Parkin. The results showed that the expression levels of PINK1 and Parkin were increased in PK-15 and 3D4/2 cells infected by CSFV at different multiplicities of infection (Figure 3A and 3B). Functionally, translocation of PINK1 and Parkin to mitochondria was well linked to the activation of mitophagy [38, 39, 41, 42]. To test whether CSFV infection induced PINK1 and Parkin accumulation in mitochondria, purified mitochondrial and cytosolic fractions of CSFV-infected and uninfected cells were analyzed by Western blotting. The results showed increased translocation of PINK1 and Parkin to purified mitochondria (Figure 3C) in PK-15 and 3D4/2 cells. Moreover, the recruitment of LC3-II to mitochondria was upregulated by CSFV infection (Figure 3C), an important marker of autophagy. This result further confirmed the formation of mitophagosomes in CSFV-infected cells.

**Figure 4:**
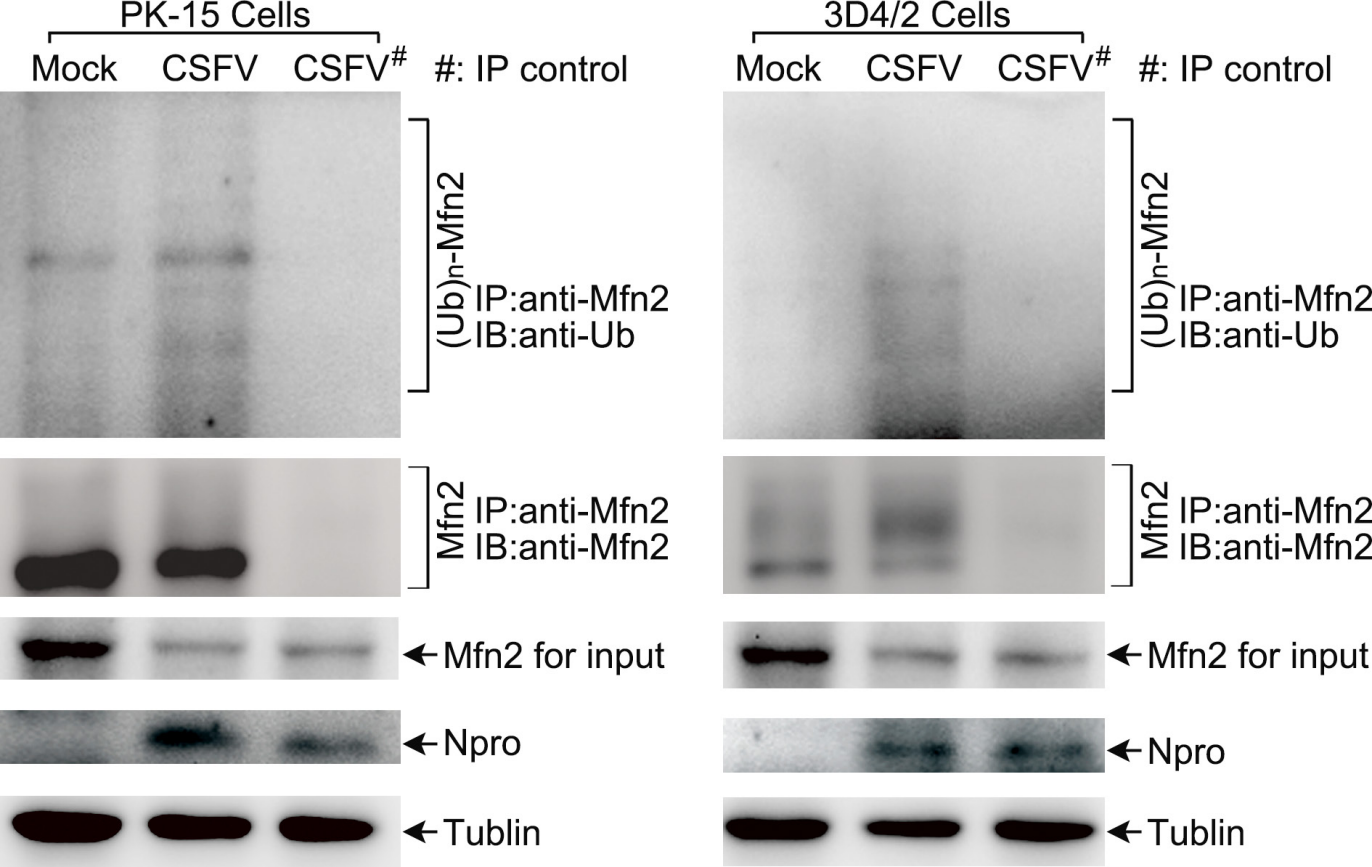
The level of MFN2 ubiquitination in CSFV-infected cells PK-15 and 3D4/2 cells were mock-infected or infected with CSFV (MOI = 1) for 48 h. The immunoprecipitation (IP) of MFN2 was confirmed by immunoblotting with anti-MFN2 antibody. The ubiquitinated MFN2 was analyzed by immunoblotting with anti-Ub antibody. Normal rabbit IgG was used as a negative control for immunoprecipitation. In addition, expression of MFN2 in CSFV-infected cells evaluated by immunoblotting was used as the input control. Tubulin was used as an internal loading control. CSFV infection was verified by immunoblotting with anti-CSFV Npro antibody.

As an E3 ubiquitin ligase, the ubiquitin of Parkin was phosphorylated by the kinase PINK1 to activate its activity and mediate ubiquitin chains on mitochondrial outer membrane proteins [49, 50]. Among multiple mitochondrial out membrane proteins, ubiquitination of MFN2 has been shown to be critical for initiating mitophagy [41, 51], whereas the role of VDAC1 on mitophagy has remained controversial [41, 52]. To detect the ubiquitination of MFN2 or VDAC1 in PK-15 and 3D4/2 cells infected by CSFV, immunoprecipitation assay was performed. As shown in Figure 4, CSFV enhanced the ubiquitination of MFN2, and the decline in MFN2 was associated with its ubiquitination (Figure 4). Because we failed to identify a suitable antibody for VDAC1 immunoprecipitation, the detection of its ubiquitination could not be performed.

### CSFV induced the formation of a mitophagosome conjunct with Parkin

**Figure 5:**
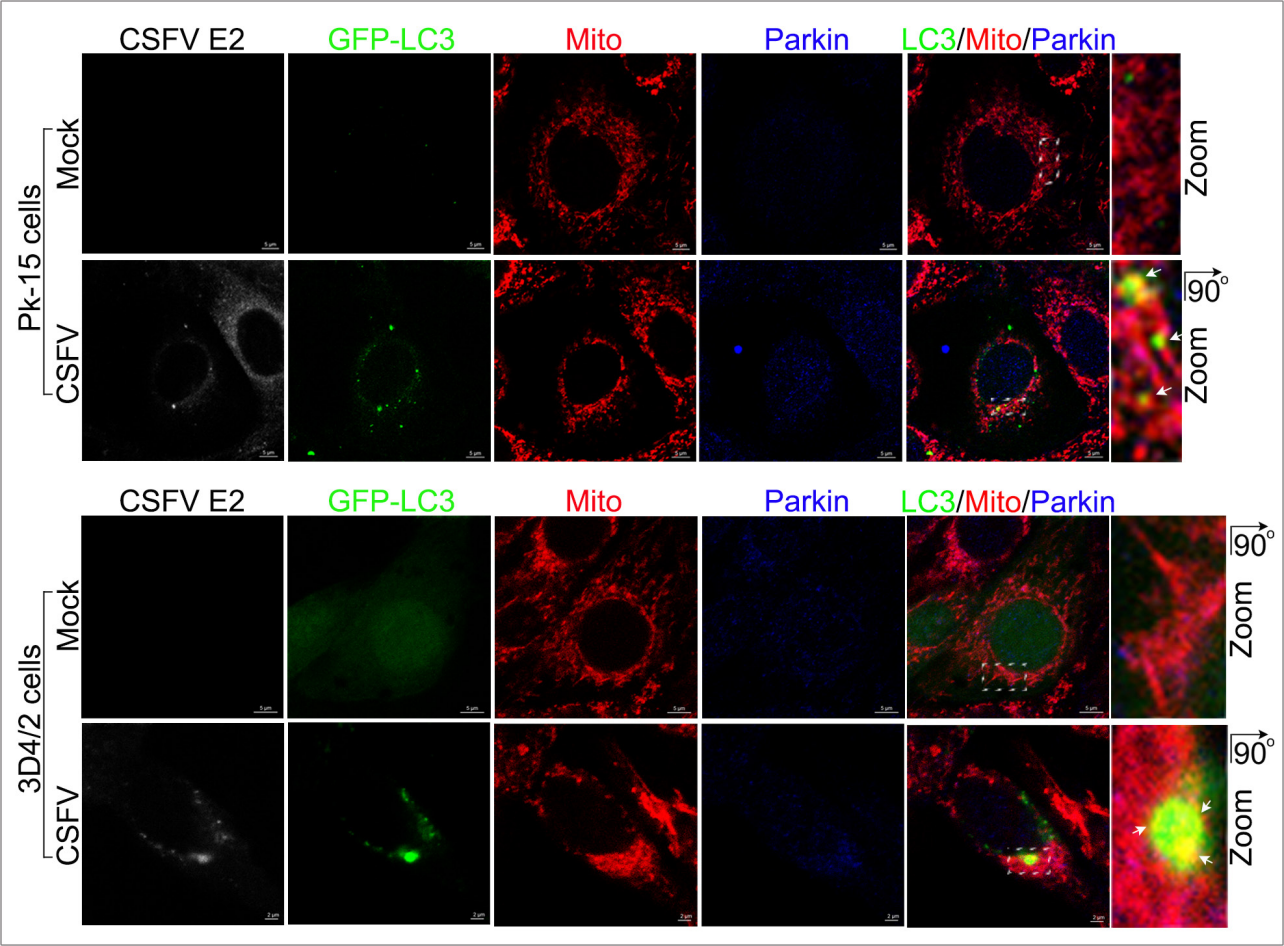
Confocal microscopy images showing mitophagosome formation associated with Parkin in CSFV-infected cells PK-15 and 3D4/2 cells transiently expressing EGFP-LC3 protein (green) were mock-infected or infected with CSFV (MOI = 1) for 48 h. After staining the mitochondria with MitoTracker (red), cells were immunostained with antibodies against CSFV E2 (white) and Parkin (blue). In the zoomed images, the arrows indicate mitochondria that were colocalized with both GFP-LC3 (yellow) and Parkin (purple).

To confirm that the mitochondria translocated by Parkin were then engulfed by autophagic phagophores, PK-15 and 3D4/2 cells transfected with GFP-LC3 were infected by CSFV and analyzed by confocal microscopy at 48 hpi. The merged images showed an increased number of mitochondria that were translocated by Parkin in CSFV-infected compared with uninfected cells (Figure 5). In addition, a partial mitochondria conjunct with GFP-LC3 puncta was associated with Parkin in PK-15 and 3D4/2 cells infected by CSFV, but not in uninfected cells (Figure 5), linking the recruitment of Parkin to mitochondria with CSFV infection-induced mitophagy.

### CSFV-induced complete mitophagy

**Figure 6:**
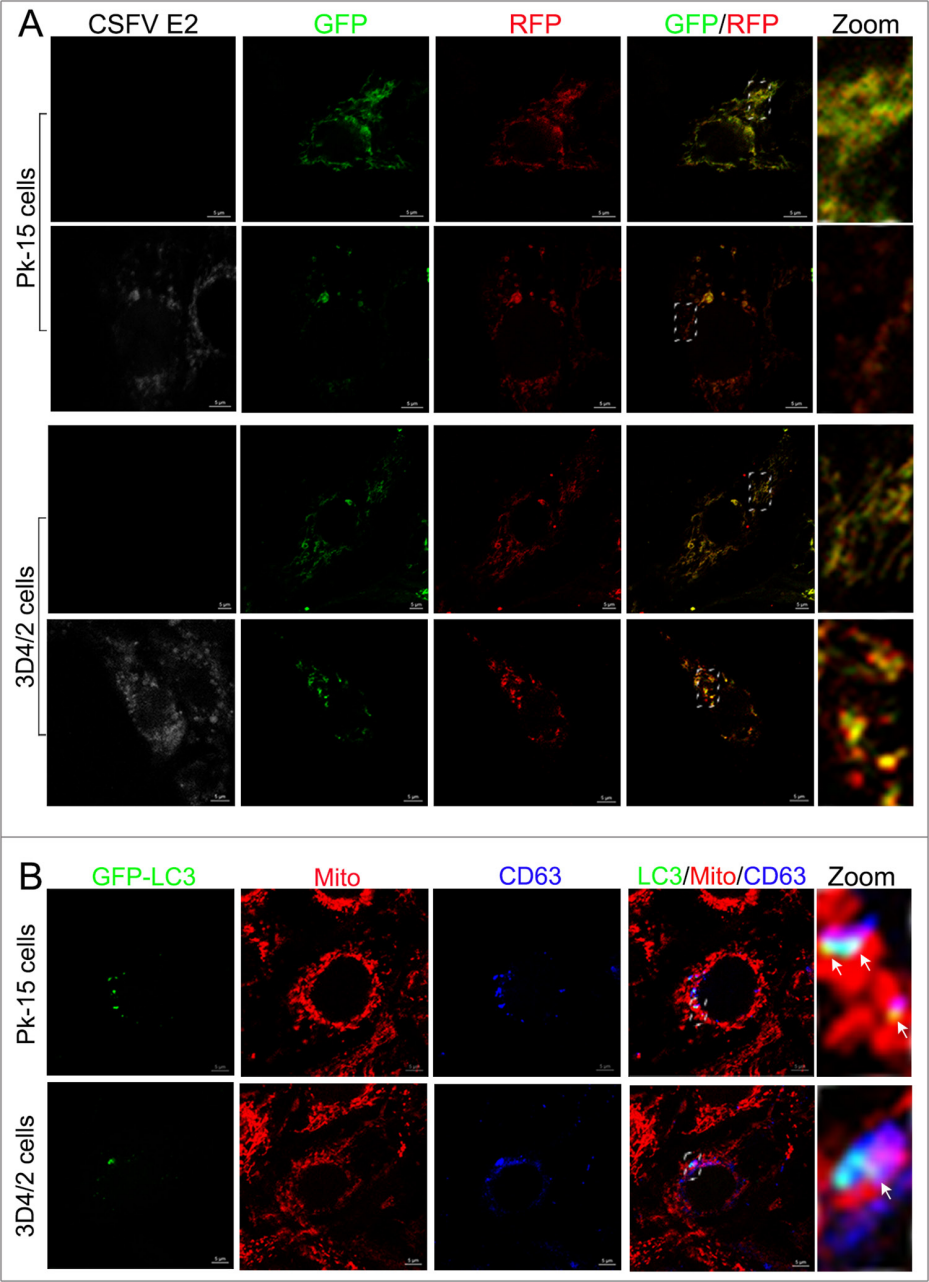
Complete mitophagy induced by CSFV infection **(A)** PK-15 and 3D4/2 cells transiently expressing Mito-mRFP-EGFP were mock-infected or infected with CSFV (MOI = 1) for 48 h. Cells were immunostained with antibodies specific to CSFV E2 (white). In the zoomed images, fluorescence signals indicated the expression of mRFP and GFP protein targeting mitochondria: yellow color, no mitophagy; red color, mitophagy. **(B)** PK-15 cells transiently expressing EGFP-LC3 protein were infected with CSFV (MOI = 1) for 48 h. After staining the mitochondria with MitoTracker (red), cells were immunostained with CD63 antibodies (blue). In the zoomed images, arrows (white puncta) show GFP-LC3 puncta (green) colocalized with mitochondria and lysosomes.

The fusion of mitophagosomes with lysosomes is a typical characteristic of complete mitophagy, in which the mitochondria are degraded and recycled by lysosomes [33]. To test whether CSFV infection caused the delivery of mitochondria to lysosomes during complete mitophagy, PK-15 and 3D4/2 cells were transfected with a tandem-tagged mRFP-EGFP plasmids encoding mitochondrial targeting signal sequences [26], which was based on the differential stabilities of mRFP and GFP in lysosomes to display mitophagy. The GFP signal is quenched at the lower pH of lysosomes, while mRFP can be consistently visualized. Thus, the preservation of red fluorescence (mRFP) indicates complete mitophagy, whereas yellow fluorescence (mRFP merged with GFP) indicates a normal mitochondrial structure. As shown in Figure 6A, in contrast to uninfected cells displaying yellow fluorescence, PK-15 and 3D4/2 cells infected by CSFV displayed more red fluorescence, indicating the degradation of mitochondria by lysosomes. To confirm the formation of mitophagolysosomes in PK-15 and 3D4/2 cells infected by CSFV, CD63, a widely used marker for lysosomes [53], was merged with GFP-LC3 and mitochondria by using confocal microscopy. The images showed that mitochondria wrapped with GFP-LC3 puncta were associated with lysosomes in CSFV-infected cells (Figure 6B).

### CSFV infection resulted in mitochondrial fission

**Figure 7:**
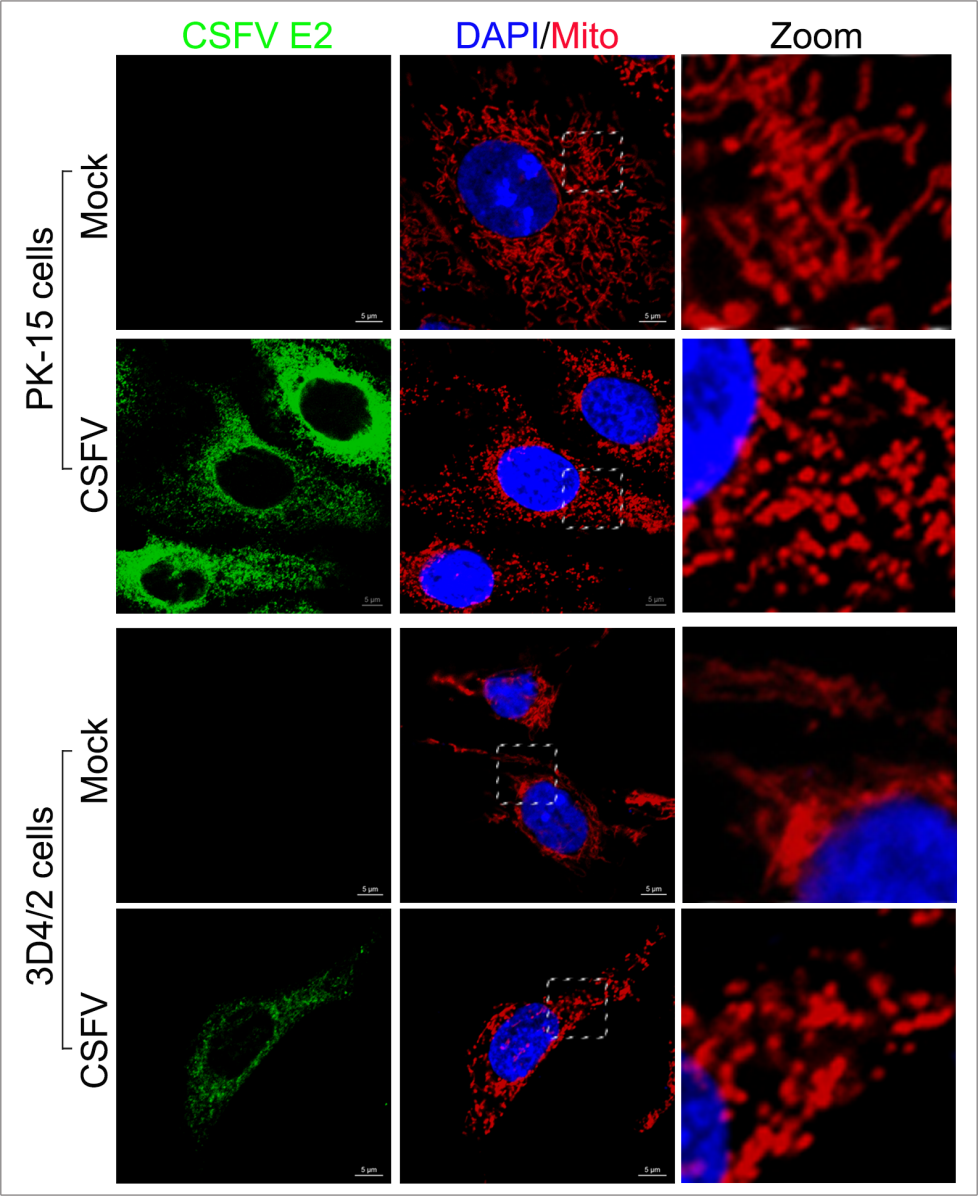
Mitochondrial fission induced by CSFV infection Confocal microscopy images showing mitochondrial fragmentation in CSFV-infected cells. PK-15 and 3D4/2 cells were mock-infected or infected with CSFV (MOI = 1). At 48 hpi, cells that had been prestained with MitoTracker (red) were immunostained with CSFV E2 antibody (green). In the zoomed images, typical tubular mitochondria in untransfected cells and fragmented mitochondria in transfected cells are shown.

**Figure 8:**
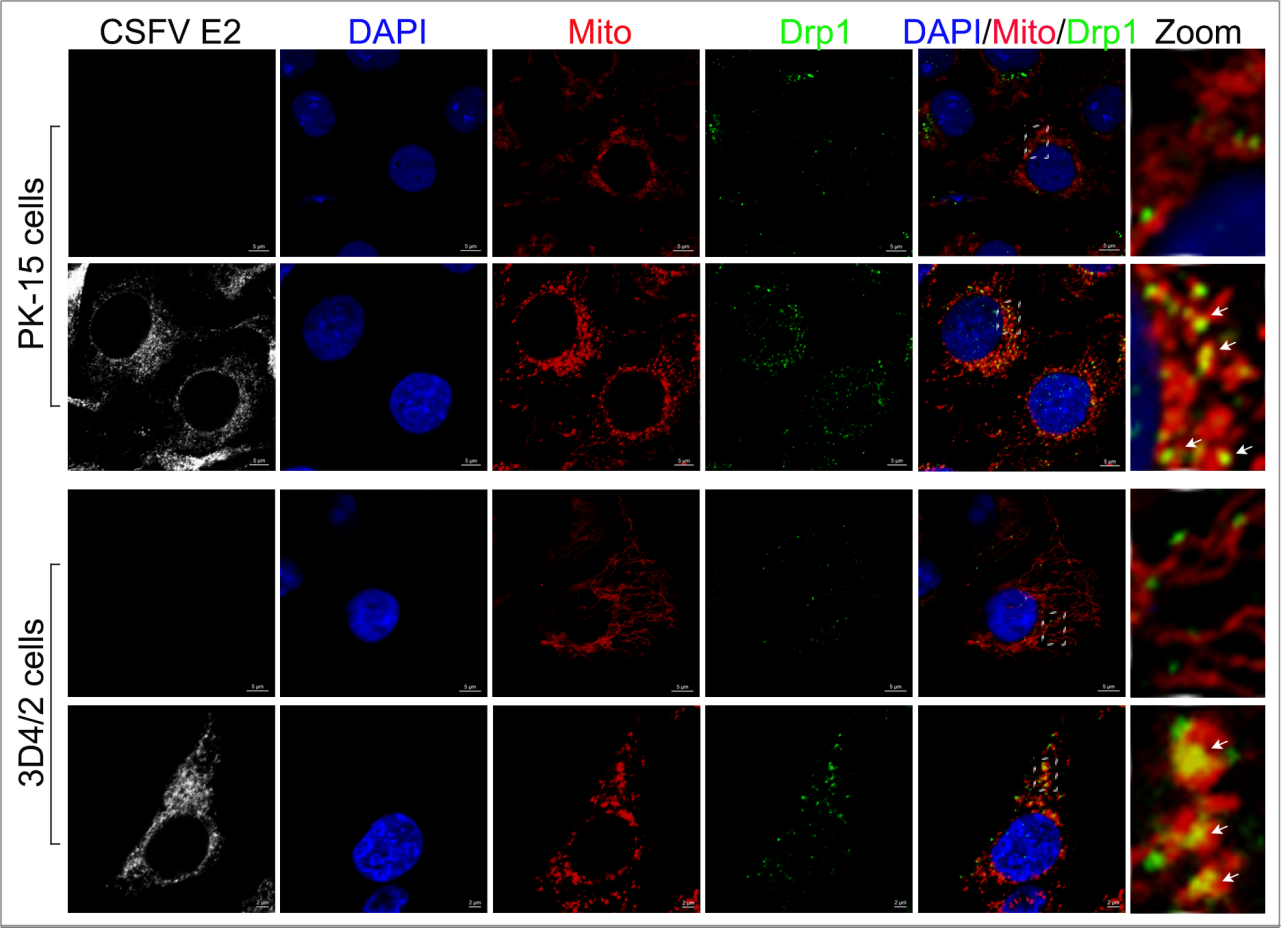
Translocation of Drp1 into mitochondria induced by CSFV infection Confocal microscopy images showing mitochondrial translocation of Drp1 in CSFV-infected cells. PK-15 and 3D4/2 cells were mock-infected or infected with CSFV (MOI = 1). At 48 hpi, cells prestained with MitoTracker (red) were immunostained with CSFV E2 antibody (white) and Drp1 antibody (green). Nuclei were stained with DAPI (blue). In the zoomed images, the arrows (yellow puncta) show the merge of Drp1 with mitochondria.

Abnormal mitochondria are usually segregated through mitochondrial fission mediated by dynamin-related protein 1 (Drp1) translocation and ultimately cleared by mitophagy [54]. It has been confirmed that HBV and HCV can disturb the balance of mitochondrial dynamics by causing mitochondrial fission followed by mitophagy [26, 28]. However, whether anomalous mitochondrial dynamics related to mitophagy occur in CSFV-infected cells has remained unclear. To observe the changes in mitochondrial dynamics in PK-15 and 3D4/2 cells infected by CSFV, a confocal immunofluorescence assay was performed. The results showed that the fragmented mitochondrial morphology was observed in CSFV-infected cells, in contrast to tubular mitochondria in uninfected cells (Figure 7). This observation was consistent with the elliptical mitochondria in TEM images of CSFV-infected cells (Figure 2A, 2A-b and 2A-d). These data indicated that CSFV infection could induce mitochondrial fragmentation. To explore whether CSFV-induced mitochondrial fragmentation was related to the translocation of Drp1 to mitochondria, colocation of Drp1 with mitochondria was analyzed by confocal microscopy. Compared with uninfected cells, an increase in merged immunofluorescence was inspected in PK-15 and 3D4/2 cells infected by CSFV (Figure 8). This result was further supported by the detection of Drp1 translocation to purified mitochondrial fractions in CSFV-infected cells (Figure 3C). Together, these results indicated that CSFV induced mitochondrial fission via Drp1 translocation.

### Interference of mitochondrial fission and mitophagy reduced CSFV replication but enhanced apoptosis

**Figure 9:**
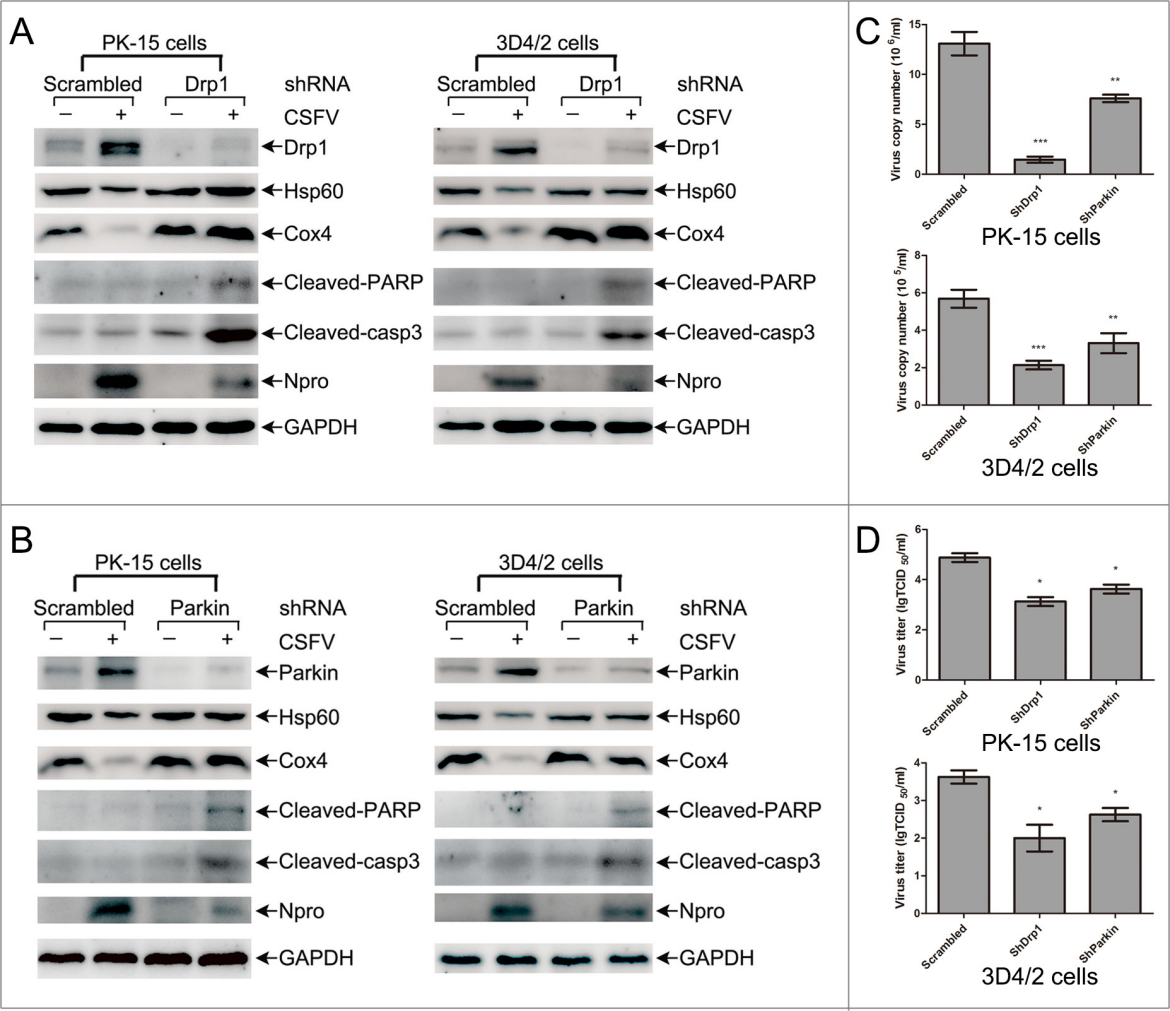
Mitochondrial proteins, replication of CSFV and apoptosis in CSFV-infected cells transfected with shRNA targeting Drp1 and Parkin **(A)** The effect of Drp1 shRNA transfection on the level of mitochondrial proteins, Npro and apoptosis in CSFV-infected cells. PK-15 and 3D4/2 cells were transfected with scrambled or Drp1 shRNA for 24 h, followed by mock infection and CSFV infection (MOI = 0.5). At 48 hpi, cells were analyzed by immunoblotting with antibodies specific to the indicated proteins. **(B)** The effect of Parkin shRNA transfection on the level of mitochondrial proteins, Npro and apoptosis in CSFV-infected cells. PK-15 and 3D4/2 cells were transfected with scrambled or Parkin shRNA for 24 h and were infected and analyzed as described in **(A)**. **(C)** Statistical analysis of the effect of Drp1 and Parkin shRNA transfection on the viral copy numbers in CSFV-infected cells. PK-15 and 3D4/2 cells were transfected with scrambled, Drp1 or Parkin shRNA for 24 h, followed by mock infection and CSFV infection (MOI = 0.5). At 48 hpi, the levels of CSFV RNA were analyzed by real-time qRT-PCR as described in Materials and Methods (mean ± SD; n = 3; **P < 0.01; ***P < 0.001). P values were calculated using an unpaired Student's t-test. **(D)** Statistical analysis of the effect of Drp1 and Parkin shRNA transfection on the virus titers in CSFV-infected cells. PK-15 and 3D4/2 cells were transfected with scrambled, Drp1 or Parkin shRNA for 24 h, followed by mock infection and CSFV infection (MOI = 0.5). At 48 hpi, the titers of CSFV were analyzed as described in Materials and Methods (mean ± SD; n = 3; *P < 0.05; ^*^P < 0.01). P values were calculated using an unpaired Student's t-test.

To assess the functional effects of mitochondrial fission and mitophagy on the course of CSFV infection, we performed shRNA knockdown experiments to silence endogenous expression of Drp1 or Parkin in PK-15 and 3D4/2 cells infected by CSFV. As shown in Figure 9A and 9B, the silencing effect on Drp1 or Parkin expression was verified by Western blotting, and changes in mitochondrial fission and mitophagy caused by the silencing of Drp1 or Parkin were checked according to the preservation of HSP60 and COX4 proteins in CSFV-infected cells. In addition, decreased expression of CSFV Npro protein in Drp1 or Parkin gene-silenced cells supported a promoting function of mitochondrial fission or mitophagy on CSFV replication. To validate this finding, the replication of CSFV in the presence of Drp1 or Parkin shRNA was analyzed by determining viral titers and RNA copies. The results indicated that Drp1 or Parkin silencing decreased viral replication in PK-15 and 3D4/2 cells (Figure 9C and 9D), indicating a positive role of mitochondrial fission and mitophagy in CSFV replication.

**Figure 10:**
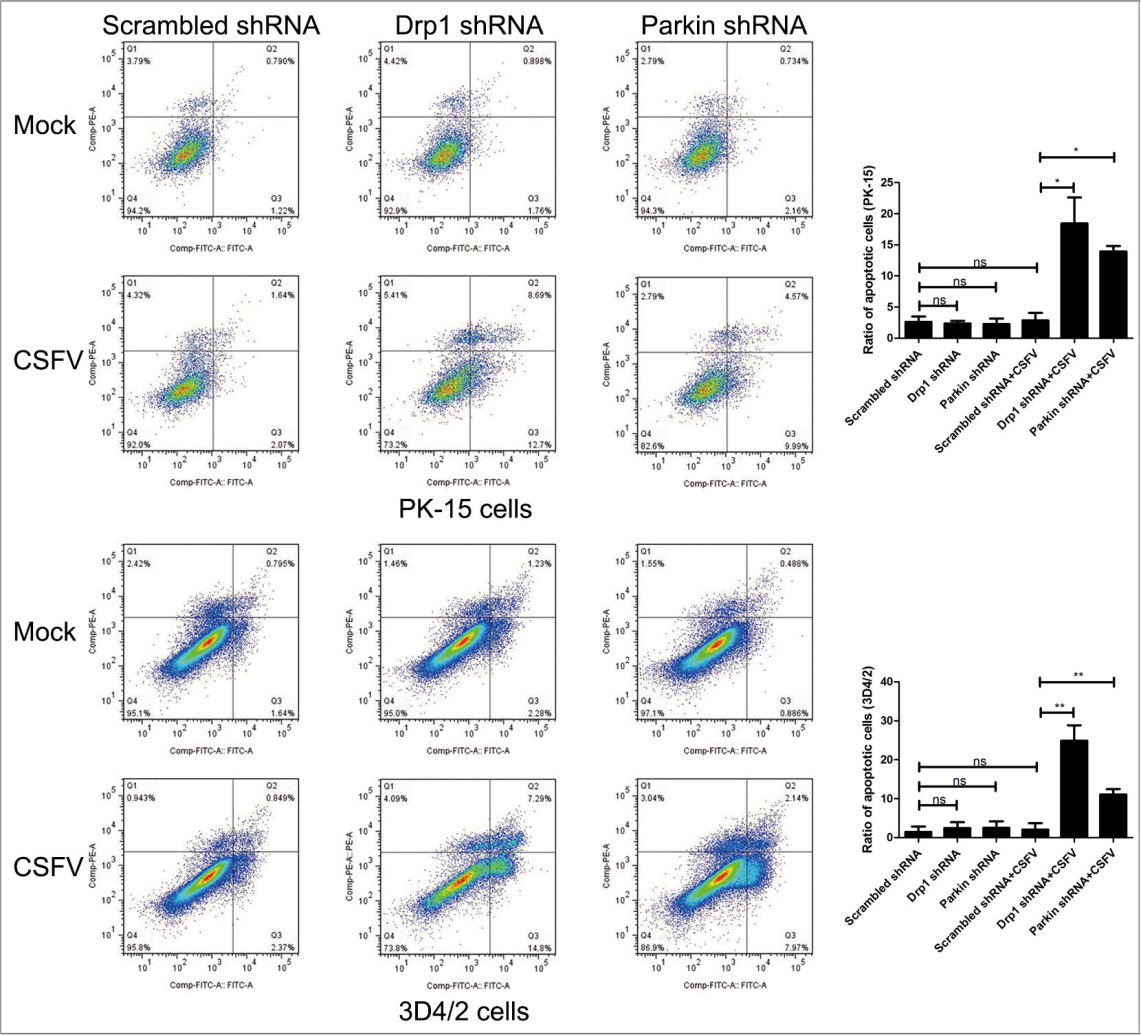
Inhibition of the expression of Drp1 or Parkin gene with target-specific shRNA promotes apoptosis of CSFV-infected cells PK-15 and 3D4/2 cells were transfected with scrambled, Drp1 or Parkin shRNA for 24 h, followed by mock infection and CSFV infection (MOI = 0.5). At 48 hpi, the rates of apoptotic cells were analyzed by using flow cytometry as described in Materials and Methods (Q1, a dead cell population that was Annexin V-negative and PI-positive. Q2, an end-stage apoptotic or a necrotic cell population that was Annexin V- and PI-positive. Q3, an early apoptotic cell population that was Annexin V-positive and PI-negative. Q4, a cell population not undergoing apoptosis that was both Annexin-V- and PI-negative). The right histograms showed the statistical analysis of the rates of apoptotic cells (cells in Q2 and Q3) (mean ± SD; n = 3; *P< 0.5; ^*^P < 0.01). P values were calculated by one-way ANOVA.

It has been previously reported that CSFV continuously blocks the apoptosis of infected cells to support viral replication [13, 14]. Because apoptosis is closely linked to mitochondrial dynamics [55, 56], we speculated that inhibition of CSFV replication in Drp1- or Parkin-silenced cells is a consequence of apoptosis related to mitochondrial fission and mitophagy. To analyze the apoptosis of CSFV-infected cells silenced by Drp1 or Parkin shRNA, cleaved-caspase-3 and cleaved-PARP were analyzed by Western blotting in PK-15 and 3D4/2 cells. The results showed that CSFV infection increased the activity of cleaved caspase-3 and cleaved PARP in cells depleted of mitochondrial fission or mitophagy (Figure 9A and 9B). Moreover, apoptosis of Drp1- or Parkin-silenced cells infected by CSFV was further confirmed by flow cytometry. The data showed that CSFV infection clearly upregulated the apoptotic rates of cells silenced by Drp1 or Parkin shRNA (Figure 10). Together, these results confirmed that CSFV prevented the apoptosis of infected cells by inducing mitochondrial fission and mitophagy to facilitate persistent viral infection.

### Cell viability was not affected by RNA interference

**Figure 11:**
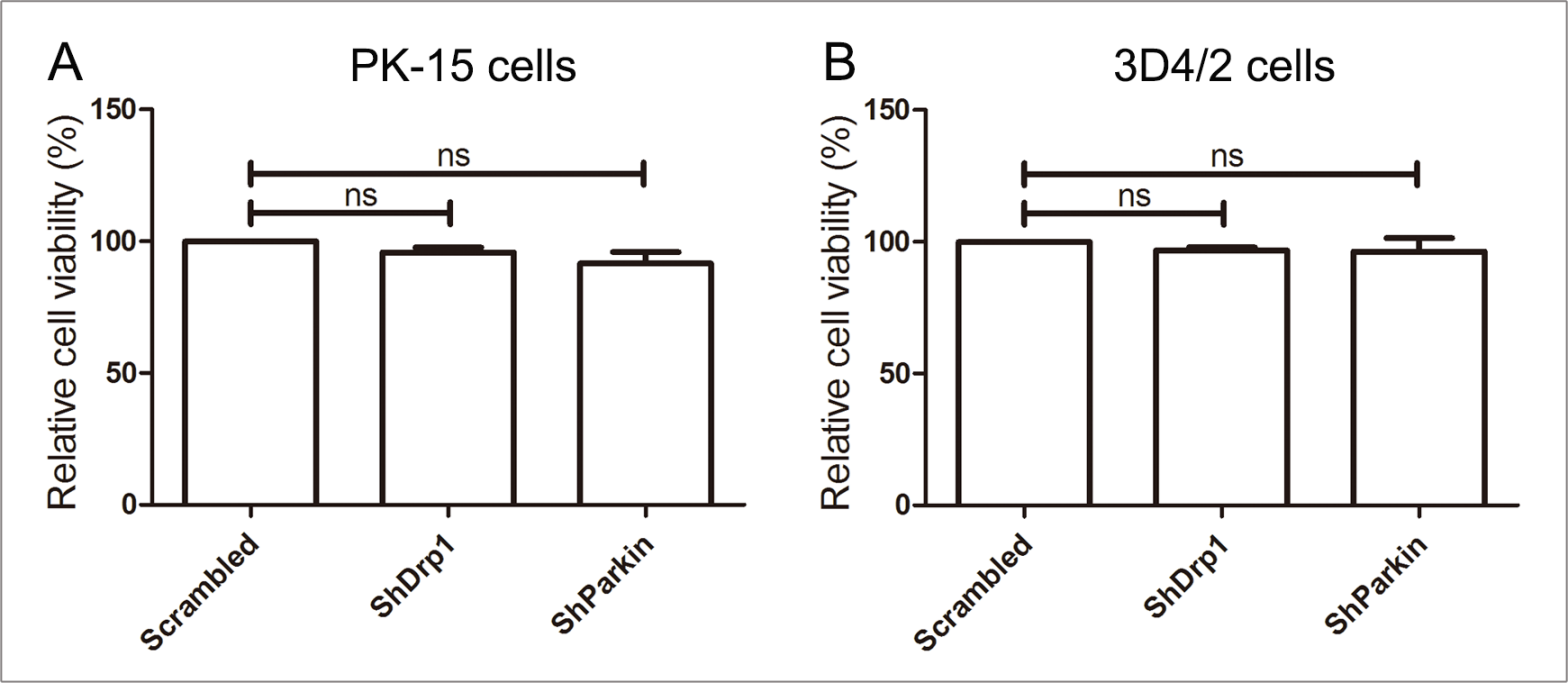
The effect of shRNA interference on cell viability The cell viability of PK-15 **(A)** and 3D4/2 **(B)** cells transfected with scrambled, Drp1 or Parkin shRNA was analyzed using the CCK8 assay as described in Materials and Methods (mean ± SD; n = 3; ^NS^P > 0.05).

To exclude the possibility that Drp1 or Parkin shRNA inhibited CSFV replication by downregulating cell viability, we analyzed the effects of RNA interference on the viability of PK-15 and 3D4/2 cells. The results showed no significant changes in the viability of cells with a silenced Drp1 or Parkin gene (P >0.05) (Figure 11).

## DISCUSSION

**Figure 12:**
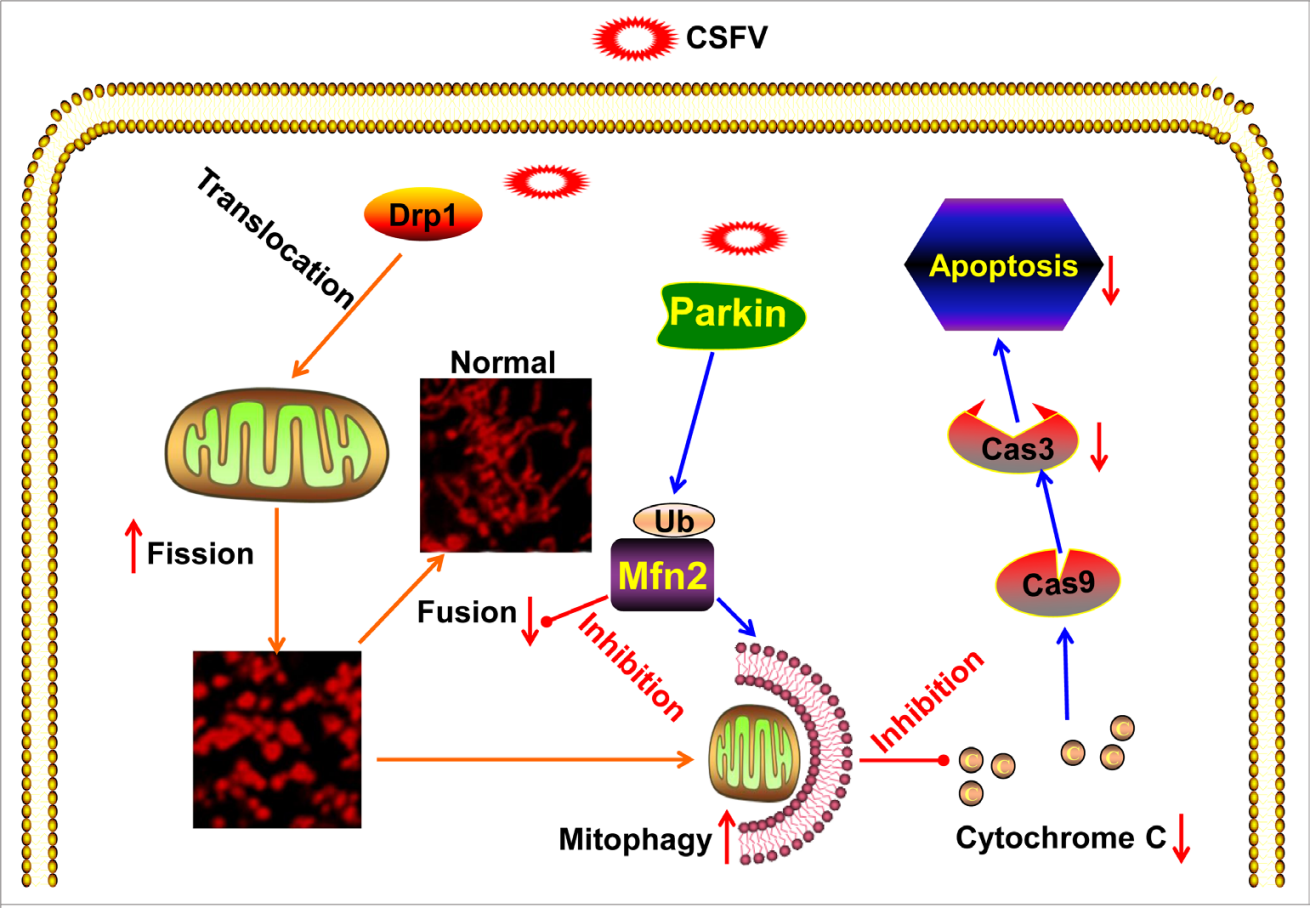
Model of CSFV induction of mitochondrial fission and mitophagy to inhibit apoptosis CSFV triggers the translocation of Drp1 and subsequent mitochondrial fission. Meanwhile, CSFV induces mitophagy to clear fragmented mitochondria by upregulating the expression and mitochondrial translocation of Parkin. Thus, mitochondrial apoptosis linked to aberrant mitochondrial fission in CSFV-infected cells is prevented via CSFV-induced mitophagy.

Viral particles are cellular parasites that only replicate in a manner that is dependent on energy sources and molecular components in living cells. Although eukaryotic cells possess systematic mechanisms that counteract viral attack, viruses have evolved corresponding countermeasures to can lead to worse responses [57]. Many viruses with noncytopathic effects can hijack various mechanisms to mitigate persistent disturbance in host cells to preserve infection [26, 28, 58]. Among the antiviral mechanisms in host cells, apoptosis and the interferon (IFN) system are particularly important for the innate immune response. Considerably, the role of mitochondria in host antiviral responses has been consistently demonstrated [59], and the characteristics of many viral proteins that localize in mitochondria and that interact with mitochondrial proteins has been revealed [24]. *In vitro*, CSFV has been shown to replicate noncytopathically within host cells. Previous reports have demonstrated that CSFV establishes long-term infection by inhibiting host cell apoptosis [13, 14]. However, the mechanisms by which CSFV regulates apoptosis have not been exhaustively explored. Apoptosis is closely linked to the perturbation of mitochondrial dynamics, including fission, fusion, and trafficking [55]. CSFV has been confirmed to induce oxidative stress *in vitro* [44–46] and to reduce mitochondrial transmembrane potential *in vivo* [10]. ROS accumulation and the collapse of mitochondrial transmembrane potential (ΔΨm) are usually due to abnormal mitochondrial dynamics during viral infection [24]. Thus, we speculate that damaged mitochondria might exist in CSFV-infected cells but that CSFV utilizes other mechanisms to remove the damaged mitochondria to inhibit cell death. It has been shown that abnormal mitochondria theoretically undergo asymmetric mitochondrial fission. Subsequently, fragmented mitochondria are removed by selective mitochondrial autophagy (mitophagy) [33]. Based on our previous study showing that autophagy enhances viral replication and the release of CSFV in host cells, we present herein, for the first time, evidence that CSFV induced mitochondrial fission and mitophagy to inhibit mitochondrion-dependent apoptosis and to promote persistent viral infection (Figure 12).

To explore the role of mitophagy in multiple steps of the CSFV life cycle or immune evasion in host cells, PK-15 and 3D4/2 cells were both used and infected by CSFV (Shimen strain). The PK-15 cell line is typically used to analyze CSFV replication and maturation [60], and 3D4/2 is a macrophage cell line that is closely related to monocytic cells for CSFV targeting infection *in vivo* [6]. Time-dependent analysis of the outer mitochondrial membrane and mitochondrial matrix proteins revealed complex changes in mitochondrial mass in PK-15 and 3D4/2 cells infected by CSFV. An increased number of mitochondria was detected at 24 hpi, whereas CSFV caused a decline in mitochondrial mass at 36~48 hpi. Similar opposing effects on the activity of mitochondria during early and later infection stages have been discovered in Herpes virus-infected cells [57]. The changes in mitochondrial mass during the life cycle of CSFV may be due to different energy needs during the progression of viral assembly. The replication of CSFV was active at 24 hpi [61], and thus an increased number of mitochondria was produced to maintain ATP levels and the rate of glycolysis during early stages of infection. At 36~48 hpi, when CSFV infection reached a relatively later stage [5], the activation of viral replication was not very strong. To validate that the decline in mitochondrial quantity was linked to CSFV-induced mitophagy, the preservation of decreased mitochondrial proteins was confirmed with the use of an autophagic inhibitor. Moreover, mitochondria trapped by double membrane vesicles, which is one of the gold standards to validate the induction of mitophagy, were observed by electron microscopy [62]. This observation was further supported by the detection of lipidated LC3B in pure mitochondrial fractions and analysis of the spatial connection of mitochondria with GFP-LC3 in CSFV-infected cells. Although many previous reports have shown that virus infection regulates the crosstalk between intracellular signaling and autophagy to enhance viral replication and maturation events [63], autophagy was only defined as a non-selective or a highly selective process according to recent findings. In contrast to non-selectively autophagic degradation of proteins and organelles for supplying cellular energy, selective autophagy consists of the recruitment of specific adaptor proteins to targets and their delivery by autophagosomes for degradation [64]. Our data offer new evidence for selective autophagy of mitochondria triggered by viral infection [31].

In general, the mechanisms of mitophagy have not been exhaustively explored in mammalian cells, and it seems that various molecules could be involved in mitophagy pathways depending on the different stimuli and cell types [32]. Notably, post-translational modifications, such as phosphorylation and ubiquitination, have been linked to the functions of adaptor proteins in selective mitophagy recognition [65]. However, the role of adaptor proteins in mitophagy remains to be fully determined [40]. Activation of the kinase PINK1 and the E3 ubiquitin ligase Parkin to remove damaged mitochondria has been linked to Hepatitis C and B virus [26, 27]. In our study, the relationship between the Pink1-Parkin pathway and CSFV-induced mitophagy was first manifested by increased expression of Pink1 and Parkin in CSFV-infected cells. Additionally, increased levels of mitochondrial translocation of Parkin analyzed by Western blotting and confocal immunofluorescence analysis certified its role in mitophagy [38]. Upregulated ubiquitination of MFN2 then further confirmed the ubiquitin ligase function of Parkin [39]. For mitochondrial fusion relevant to MFN2 function, the degradation of its ubiquitin by Parkin will result in mitochondrial fission [51]. Finally, the relationship between Parkin translocation to mitochondria and selective mitophagy was checked by observing merged images of Parkin, GFP-LC3 and Mitotracker using a confocal microscope. A previous study has shown that mutations in Parkin are linked to Parkinson's disease, a neurodegenerative disorder, highlighting the crucial function of mitophagy in mitochondrial homeostasis and cell survival [34].

Diverse independent reports have confirmed the controversial role of incomplete and complete autophagy in the viral life cycle [63]. Our previous data have shown that complete autophagic responses are triggered by CSFV infection in host cells, and the degradation of a small amount of viral particles by lysosomes is inhibited by E64d treatment [47]. Here, we demonstrated complete mitophagy by colocalization analysis of mitophagosomes with lysosomes and a dual fluorescence reporter (Mito-mRFP-EGFP) in CSFV-infected cells. This finding is consistent with several recent reports demonstrating complete mitophagy in virus-infected cells [31].

It has been proposed that asymmetric fragmentation of mitochondria provides better substrates for mitophagy, whereas fused mitochondria cannot be cleared by selective autophagy [33]. Based on our present data showing that CSFV induces complete mitophagy to maintain mitochondrial homeostasis in host cells, we speculated that mitochondrial fission may occur in CSFV-infected cells. Interestingly, fragmentation of mitochondria was indeed inspected using electron microscopy and confocal microscopy. By detecting increased enrichment of Drp1on mitochondria in host cells infected by CSFV, this speculation was further confirmed. CSFV infection has been previously linked to mitochondrial dysfunction relevant to oxidative stress and altered the mitochondrial transmembrane potential [10, 44]. Here, we discovered that CSFV induced mitochondrial fission and the clearance of damaged mitochondria by mitophagy. The fission of abnormal mitochondria and subsequent clearance of mitochondrial fragmentation via mitophagy is crucial for the maintenance of mitochondrial homeostasis and cell survival [20, 33].

Because mitochondria exert a crucial function on many cellular processes and have been continuously linked to antiviral responses [57], we attempted to reveal the significance of mitochondrial fission and mitophagy in CSFV-infected cells. It is worth noting that Drp1 and Parkin silencing affected viral multiplication, which is consistent with our previous report describing autophagy during CSFV replication [47]. Most importantly, apoptosis of CSFV-infected cells was upregulated when mitochondrial fission or mitophagy was interrupted by the corresponding shRNA. As a noncytopathic virus, CSFV inhibits the apoptosis of host cells to maintain long-term infection [13, 14]. ROS that are mainly produced by damaged mitochondria can continuously cause subsequent damage to healthy mitochondria and ultimately lead to cell death. To a certain degree, the rapid clearance of damaged mitochondria demonstrated by our findings illustrated that CSFV induced ROS accumulation and inhibited apoptosis concurrently in host cells [13, 14]. Moreover, it has been previously reported that CSFV inhibits interferon synthesis during *in vitro* infection [13, 61]. Additionally, a recent study showed that HCV infection triggers mitochondrial fission and mitophagy to attenuate interferon responses [28]. Whether the disruption of mitochondrial dynamics by CSFV is relevant to immune evasion against interferon in host cells remains to be explored.

In summary, our study offers the first evidence that CSFV takes advantage of mitochondrial fission and mitophagy to enhance persistent infection. The inhibition of apoptosis possibly mediated by CSFV-induced altered mitochondrial dynamics in host cells, especially in 3D4/2 cells, is an explication of persistent infection and high viral loads in monocytic cells of pigs infected by CSFV [6]. Monocytic cells play critical role in immunopathology, and thus the long-term presence of viral particles in these cells is likely to be connected to immunosuppression and the hemorrhage of CSFV-infected piglets [15]. Overall, the abnormal mitochondrial dynamics and selective mitophagy observed herein provide a unique view of the pathogenesis of CSFV infection and a new route of thinking for the development of antiviral strategies.

## MATERIALS AND METHODS

### Cell culture and virus

The swine kidney cell line PK-15 (ATCC, CCL-33) and porcine macrophage cell line 3D4/2 (ATCC, CRL-2845) were cultured as described previously [47]. The CSFV strain (Shimen) used in the study was prepared and titered as described previously [47]. For different experiments, viral infection was carried out at a multiplicity of infection (MOI) of 1 for mitophagy induction and an MOI of 0.5 for viral replication studies.

### DNA constructs

Plasmid pAT016 (p-mito-mRFP-EGFP) was a kind gift from Dr. Andreas Till (University of California, United States of America). Plasmid pEGFP-C1 was purchased from Clontech. Plasmid pEGFP-LC3 was constructed as previously described [66]. Briefly, cDNA encoding porcine LC3-II was obtained by RT-PCR from total RNA of the porcine macrophage cell line with the following primers: LC3-F (5′-GGCTGAGGAG ACACAAGAGA-3′) and LC3-R (5′-CAAAGCTGAA TGTGCTCGTC-3′). LC3-II cDNA lacking the termination codon was then inserted into the BglII and HindIII sites of pEGFP-C1. Additionally, 3 specific shRNAs, respectively, targeting Drp1 and Parkin, along with the scrambled shRNA, were designed by and obtained from Cyagen. The protein targeted for knockdown was evaluated by Western blotting. The shRNA sequence used in this study was as follows. shDrp1 sequence: GGAGTAAGCC CTGAACCAAT CTCTTGAATT GGT TCAGGGC TTACTCC. shParkin sequence: GCATCACCTG TACGGACATT CTCTTGAAAT GTCCGTACAG GTGATGC.

### Reagents and antibodies

The chemical reagents 3-methyladenine (M9281) and Bafilomycin A1 (B1793) were purchased from Sigma-Aldrich. The primary antibodies used in this study were as follows: rabbit polyclonal anti-PARK2 (Abnova, PAB0714); rabbit polyclonal anti-LC3B (Cell Signaling, 2775); rabbit polyclonal anti-HSP60 (Abclonal, A0969); rabbit polyclonal anti-Caspase-3 (Abclonal, A2156); rabbit polyclonal anti-COX4 (Proteintech, 11242-1-AP); rabbit polyclonal anti-MFN2 (Santa Cruz, sc-50331); rabbit polyclonal anti-CD63 (Santa Cruz, sc-15363); goat polyclonal anti-VDAC1 (Santa Cruz, sc-32063); goat polyclonal anti-Tom20 (Santa Cruz, sc- 11021); rabbit polyclonal anti-SQSTM1/P62 (Sigma, SAB2104334); mouse monoclonal anti-Ubiquitin (Cell Signaling, 3936); mouse monoclonal anti-Drp1 (Cell Signaling, 8570); mouse monoclonal anti-CSFV E2 (JBT, 9011); rabbit polyclonal anti-PARP (Beyotime, AP102); mouse monoclonal anti-GAPDH (Beyotime, AG019); mouse monoclonal anti-Tubulin (Beyotime, AT819); normal rabbit IgG (Beyotime, A7016); normal goat IgG (Beyotime, A7007); mouse polyclonal anti-CSFV N^pro^ (kindly provided by Dr. Xinglong Yu, Veterinary Department, Hunan Agricultural University, China). The secondary antibodies used for immunofluorescence were Alexa350 goat anti-mouse IgG (Beyotime, A0412), Alexa488 goat anti-mouse IgG (Beyotime, A0428) and Alexa647 goat anti-rabbit IgG (Beyotime, A0468). The secondary antibodies used for immunoblotting analysis were HRP-conjugated goat anti-mouse IgG (Bioworld Technology, BS12478), HRP-conjugated goat anti-rabbit IgG (Bioworld Technology, BS13278) and HRP-conjugated rabbit anti-goat IgG (Bioworld Technology, BS30503).

### Biochemical intervention

To inhibit autophagic flux, PK-15 or 3D4/2 cells grown to 80% confluence in 12-well cell culture plates were pretreated with 5 nM Bafilomycin A1 for 4 h. After CSFV infection, the cells were cultured in fresh medium containing 5 nM Bafilomycin A1 for 48 h. To inhibit the induction of autophagy, PK-15 or 3D4/2 cells grown to 80% confluence in 12-well cell culture plates were pretreated with 5 mM 3-MA for 4 h prior to viral infection. The mock-infected or CSFV-infected cells were then incubated in fresh medium containing 5 mM 3-MA for 48 h. Simultaneously, the same amount of dimethyl sulfoxide (DMSO) was added as control.

### Transfection and gene silencing with shRNA

PK-15 or 3D4/2 cells grown to 80% confluence in 12-well cell culture plates were transfected with Drp1, Parkin or non-targeting shRNA using the Lipofectamine® 3000 reagent (ThermoFisher, L3000015). Briefly, 1 μg of shRNA and 2 μL P3000 was diluted in 50 μl of serum-free OptiMEM medium, and 3 μL Lip3000 was also diluted in 50 μl of serum-free OptiMEM medium. The dilutions were completely mixed and incubated at 25°C for 5 min. The mixture was then pipetted into the medium and further cultured at 37°C for 24 h. Following CSFV infection, the cells were incubated in fresh medium at 37°C for 48 h.

### Cell viability assay

Cell viability was analyzed using the CCK8 assay according to the manufacturer's instructions (Dojindo, CK04). Briefly, PK-15 and 3D4/2 cells were seeded in 96-well culture plates at a density of 1 × 10^4^ cells per well and cultured for 24 h at 37°C. The cells were transfected with Drp1, Parkin or non-targeting shRNA using Lipofectamine® 3000 reagent. After 48 h, the medium was replaced with 100 μl of fresh medium containing 10 μl of CCK8. The cells were further cultured for 1 h at 37°C, and the optical density was measured at 570 nm using a model 680 microplate reader (Bio-Rad).

### Isolation of mitochondria

PK-15 or 3D4/2 cells grown to 80% confluence in 10-cm dishes were infected by CSFV and further cultured for 48 h. After washing twice with PBS, the cells were dispersed with trypsin, collected by centrifugation, and the cell suspension was harvested at approximately 850 g for 2 min. Mitochondrial isolation of PK-15 or 3D4/2 cells was performed using reagent-based methods according to the manufacturer's instructions (ThermoFisher, 89874).

### Electron microscopy

PK-15 or 3D4/2 cells grown in 10-cm dishes were washed twice with PBS and fixed with 2.5% glutaraldehyde diluted in PBS at 4°C for 30 min. The cells were then collected in 1.5-ml Eppendorf tubes and further fixed overnight. Cell pellets were dehydrated with an acetone series and embedded in epoxy resin. Next, ultrathin sections were prepared and observed using a JEM-2010HR TEM (JEOL).

### Confocal immunofluorescence microscopy

PK-15 or 3D4/2 cells were grown in 35-mm petri dishes (NEST, GBD-35-20) with a glass bottom. When needed, the indicated plasmid DNA was transfected prior to CSFV infection. Mitochondria in live cells were stained with 100 mM MitoTracker CMXRos Red (Invitrogen) for 30 min at 37°C. After washing twice with PBS and fixation with 4% paraformaldehyde for 30 min, the cells were permeabilized with 0.2% Triton X-100 for 10 min. The 5% bovine serum albumin (BSA) dissolved in PBS was used to block the cells. Next, the cells were stained with the indicated primary antibodies and appropriate secondary antibodies for 1 h at 37°C. Wherever indicated, nuclei were stained with DAPI (Beyotime). Images were obtained under a 100 × oil objective using an LSM710 (Leica) confocal microscope. Image quantification was performed with Image-Pro Plus 6.0.

### Real-time RT-qPCR

Total RNA was prepared using the Total RNA Kit I (Omega, R6834-01), and complementary DNAs (cDNAs) were synthesized using PrimeScript™ RT Master Mix (TAKARA, RR036A) according to the manufacturer's instructions. Real-time qPCR was performed using SYBR® Premix Ex Taq™ II (TAKARA, RR820A) on an iQ5 iCycler detection system (Bio-Rad). The CSFV-specific primers and reaction conditions have been described previously [47].

### Immunoblotting

Cells were incubated on ice with RIPA lysis buffer (Beyotime, P0013B) containing 1 mM PMSF (Beyotime, ST506) for 10 min. The protein concentration was quantified by the BCA protein assay kit (ThermoFisher, 23227). Equal amounts of proteins (20 μg) were separated on 15% SDS-PAGE gels and then electrotransferred onto polyvinylidene fluoride (PVDF) membranes (Millipore, IPVH00010). The 5% nonfat milk dissolved in PBS containing 0.1% Tween 20 was used to block the membranes for 1 h at 25°C. The membranes were then incubated with the corresponding primary antibodies at 4°C overnight and the secondary antibodies conjugated to HRP at 37°C for 1 h. The bands were detected using the ECL Plus kit (Beyotime, P0018) and imaged using a chemiluminescence imaging system (Fine-do X6, Tanon). Protein blots were measured with Image-Pro Plus 6.0 software.

### Immunoprecipitation

For immunoprecipitation of the ubiquitinated MFN2 or VDAC1 in the whole cell lysates (WCL), cells were incubated on ice with IP lysis buffer (Beyotime, P0013) containing 1 mM PMSF (Beyotime, ST506) for 10 min. The precipitates were removed by centrifugation at 14,000 g for 10 min at 4°C. The supernatant was immunoprecipitated with the appropriate antibodies (anti-MFN2 or anti-VDAC1) and protein A+G Sepharose (7sea biotech, P001-2). The immunoprecipitated proteins were then analyzed by Western blotting with ubiquitin antibodies.

### FACS analysis

After washing twice with PBS, the cells were dispersed by trypsin and collected by centrifugation of the harvested cell suspension at approximately 850 g for 2 min. Apoptosis of the cells was performed using the Annexin V-EGFP Apoptosis Detection Kit I (Vazyme, A212-01/02) according to the manufacturer's instructions.

### Statistical analysis

Statistical analysis was performed using the unpaired Student's t-test utilizing GraphPad Prism 5 software.

## SUPPLEMENTARY MATERIALS FIGURES AND TABLES



## Abbreviations

3-MA: 3-methyladenine
BafA1: Bafilomycin A1
CSFV: classical swine fever virus
DAPI: 4′ 6-diamidino-2-phenylindole
EGFP: enhanced green fluorescent protein
FBS: fetal bovine serum
hpi: hours post-infection
HRP: horseradish peroxidase
LC3: microtubule-associated protein 1 light chain 3
MOI: multiplicity of infection
PBS: phosphate-buffered saline
qRT-PCR: real-time quantitative reverse transcriptase polymerase chain reaction
ROS: reactive oxygen species
shRNA: short hairpin RNA
TCID_50_: 50% tissue culture infectious doses
TEM: transmission electron microscopy
