# Supplementary material for: CSFV induced mitochondrial fission and mitophagy to inhibit apoptosis
Source: Oncotarget. 2017 Apr 11;8(24):39382–400. doi: 10.18632/oncotarget.17030 (PMC5503620; doi:10.18632/oncotarget.17030)
Supplement: Supplementary file 1 [file oncotarget-08-39382-s001.pdf]

CSFV induced mitochondrial fission and mitophagy to inhibit apoptosis

SUPPLEMENTARY FIGURE

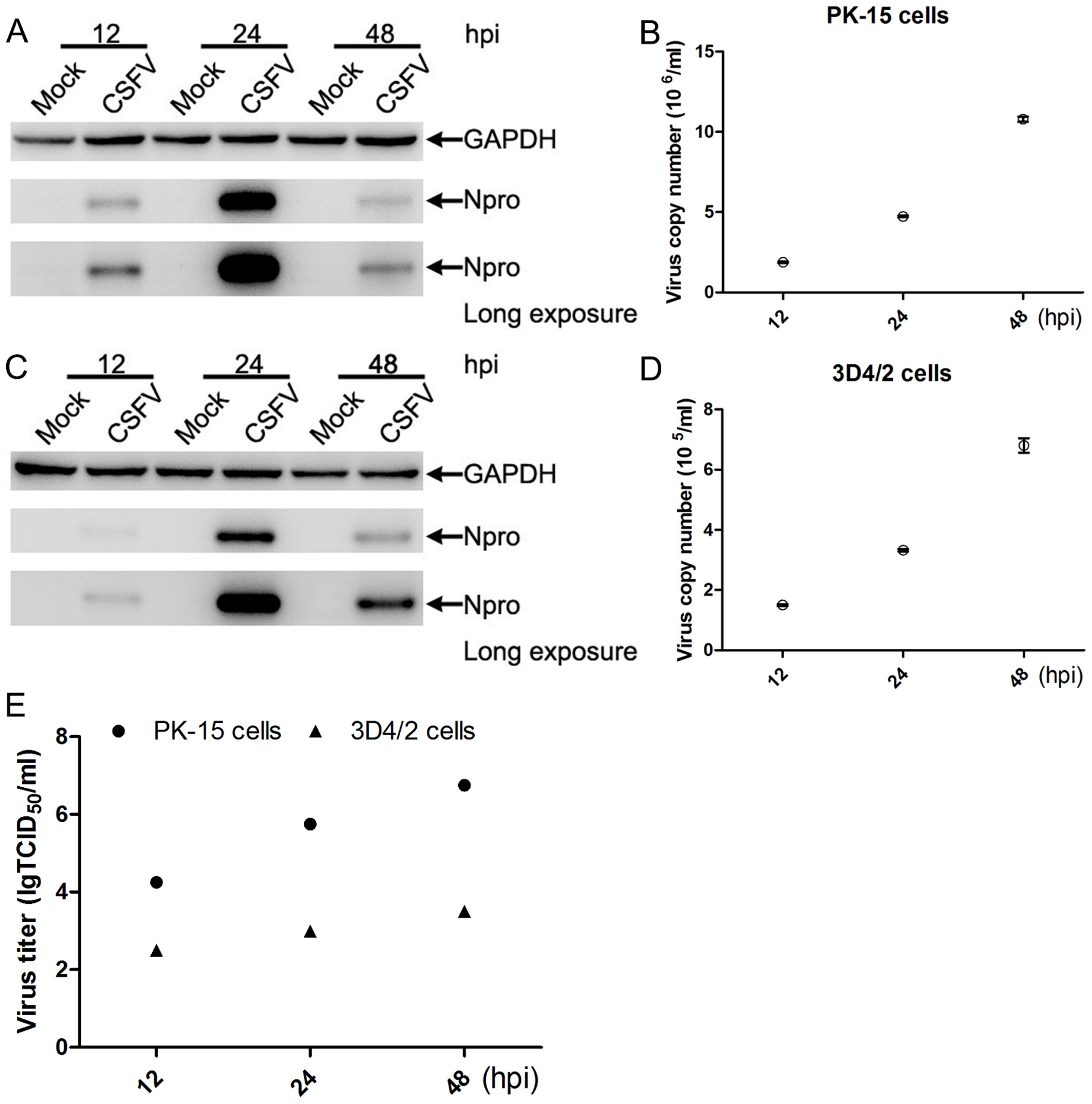

Supplementary Figure 1: Relation of Npro protein and viral replication in CSFV-infected cells. **(A and C)** Expression of Npro protein in CSFV-infected PK-15 and 3D4/2 cells. PK-15 and 3D4/2 cells were mock-infected or infected with CSFV (MOI = 1), and whole cell lysates (WCL) were prepared at 12, 24 and 48 hpi. The expression of Npro protein was analyzed by Western blotting. **(B and D)** Statistical analysis of viral copy numbers in CSFV-infected PK-15 and 3D4/2 cells. PK-15 and 3D4/2 cells were mock-infected or infected with CSFV (MOI = 1). At 12, 24 and 48 hpi, the levels of CSFV RNA were analyzed by real-time qRT-PCR as described in Materials and Methods (mean ± SD; n = 3). **(E)** Viral titers in CSFV-infected PK-15 and 3D4/2 cells. PK-15 and 3D4/2 cells were mock-infected or infected with CSFV (MOI = 1). The titers of CSFV were analyzed as described in Materials and Methods.
